# Daily Lipolysis Gene Expression in Male Rat Mesenteric Adipose Tissue: Obesity and Melatonin Effects

**DOI:** 10.3390/ijms26020577

**Published:** 2025-01-11

**Authors:** Pilar Cano-Barquilla, Vanesa Jiménez-Ortega, Pilar Fernández-Mateos, Leire Virto, Estela Maldonado Bautista, Juliana Perez-Miguelsanz, Ana I. Esquifino

**Affiliations:** 1 Departamento de Bioquímica y Biología Molecular, Facultad de Medicina, Universidad Complutense de Madrid, 28040 Madrid, Spain; 2 Instituto de Investigación Sanitaria del Hospital Clínico San Carlos (IdISSC), 28003 Madrid, Spain; lvirto@ucm.es (L.V.); jperezm@ucm.es (J.P.-M.); 3 Departamento de Biología Celular e Histología, Facultad de Medicina, Universidad Complutense de Madrid, 28040 Madrid, Spain; 4 Departamento de Anatomía y Embriología, Facultad de Óptica y Optometría, Universidad Complutense de Madrid, 28037 Madrid, Spain; 5 Departamento de Anatomía y Embriología, Faculta de Medicina, Universidad Complutense de Madrid, 28040 Madrid, Spain; emaldonado@ucm.es

**Keywords:** obesity, melatonin, lipolysis, mesenteric adipose tissue, rats

## Abstract

Melatonin is involved in various functions such as the timing of circadian rhythms, energy metabolism, and body mass gain in experimental animals. However, its effects on adipose tissue lipid metabolism are still unclear. This study analyzes the effects of melatonin on the relative gene expression of lipolytic proteins in rat mesenteric adipose tissue and free fatty acid (FFA) and glycerol plasma levels of male Wistar rats fed a high-fat (HFD) or maintenance diet. Four experimental groups were established: control, obese, and control or obese plus 2.3 mg/kg/day of melatonin in tap water. After 11 weeks, animals were sacrificed at different times throughout a 24 h cycle, and mesenteric adipose tissue and plasma samples were collected and analyzed. *Cgi58*, *Perilipin*, and *Dgat1* gene expression, as well as FFA and glycerol concentrations, showed rhythm patterns in the control group. HFD disrupted those rhythm patterns and increased FFA and glycerol concentrations during the dark photoperiod. In both melatonin-treated groups, almost all analyzed genes showed circadian patterns. Notably, melatonin significantly prevented the increase in FFA levels during the dark photoperiod in obese rats (obese group: ~1100 mM vs. obese + melatonin group: ~600 μM, similar to control levels). However, the rhythmic pattern observed in control animals was not sustained. According to our results, melatonin could regulate circadian gene transcription of mesenteric adipose tissue lipolysis proteins. The effect of melatonin on preventing elevated FFA plasma levels associated with high-fat diet intake warrants further investigation.

## 1. Introduction

Adipose tissue includes different functionally distinct anatomical depots that independently relate to the underlying metabolic complications of obesity. White adipose tis-sue is divided into two major deposits: subcutaneous adipose tissue (SAT) and visceral adipose tissue (VAT), which is associated with the abdominal and internal thoracic organs (See [1,2,3] for review). Within this tissue there are unilocular adipocytes involved in functions that show strong variations throughout the day, such as the secretion of adipokines or the storage and mobilization of fatty acids [4,5]. After food intake (during the active/wake phase), adipocytes synthesize triacylglycerides (TAG) and store them in lipid droplets (LD). During fasting (inactive/sleep phase), TAGs are metabolized into their free fatty acids (FFA) and glycerol components via the metabolic pathway called neutral lipolysis. The products of lipolysis are then released into the circulatory system to supply the energy demands of other tissues [6].

Neutral lipolysis involves three lipases acting consecutively: adipose triglyceride lipase (ATGL) or patatin-like phospholipase domain containing-2 (PNPLA2) (the recommended name) [7], hormone-sensitive lipase (HSL), and monoacylglycerol lipase (MGL) [8].

**Figure 1:**
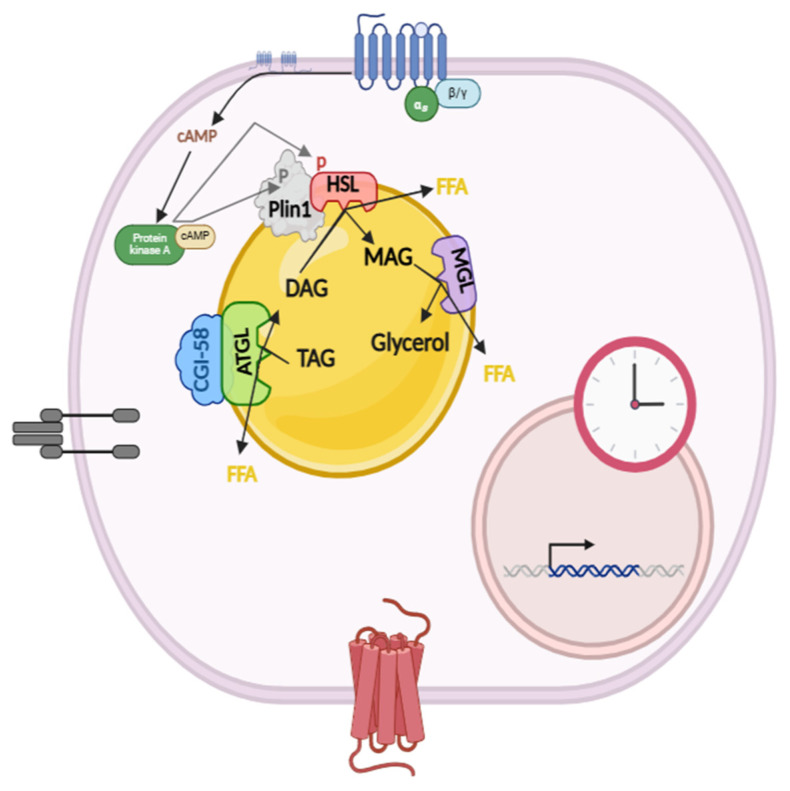
At the post-transcriptional level, pro- and anti-lipolytic hormones, such as catecholamines and insulin, directly control lipolysis activity through regulation of intracellular cAMP levels (Created in BioRender.com, accessed on 20 June 2024). AC: adenylate cyclase, Plin 1: perilipin 1.

ATGL is the rate-limiting enzyme of this process and catalyzes the initial step of cleaving TAG into diacylglycerol (DAG) and FFAs [9]. Its activity is regulated by different proteins such as comparative gene identification-58 (CGI-58), also called α/β hydrolase domain-5 (ABHD5) [8,10,11]. On the other hand, hormone-sensitive lipase (HSL) hydrolyzes DAGs to monoacylglycerols (MAGS) and FFAs, but also acts on other substrates. HSL, once phosphorylated, increases its translocation from the cytosol to the LD and interacts with different LD-associated proteins, mainly perilipins, becoming activated [12,13]. MGL (monoglyceride lipase) finally cleaves MAGS into glycerol and FFA (Figure 1).

A significant proportion of lipolyzed fatty acids are re-esterified into triacylglycerols (TAGs) by acyl-CoA: diacylglycerol acyltransferase (DGAT) enzymes during fasting (inactive/sleep phase). There are two distinct and unrelated isozymes, DGAT1 and DGAT2, that catalyze this biochemical reaction and together are responsible for the majority of TAG synthesis [14].

Lipolysis is regulated by endocrine, paracrine, and autocrine factors (hormones, cytokines, and neurotransmitters) at multiple levels, including gene transcription and enzyme activity. [8,15,16,17]. This process is therefore not solely dependent on food intake or physical activity [18,19]. The circadian system, a biological mechanism adapting physiological processes to environmental cycles (e.g., light–dark cycles, temperature, and nutrient availability), also plays a crucial role [20]. This system is controlled by the suprachiasmatic nucleus (SCN), the master circadian pacemaker, which synchronizes peripheral clocks in organs and tissues [21,22]. Molecular feedback loops involving the circadian locomotor output cycles kaput (CLOCK) and the muscle aryl hydrocarbon receptor nuclear translocator-like protein 1 (BMAL1) proteins within these clocks [21] regulate gene expression [23], including genes involved in lipolysis (e.g., *Atgl* and *Hsl* in mouse white adipose tissue) [18,24,25]. However, chrono-disruptors, such as altering the timing of food intake or diet composition, can desynchronize these clocks and have been associated with an increased risk of developing metabolic diseases [26,27].

Melatonin, an endogenous indoleamine hormone, is rhythmically synthesized in the pineal gland [28]. In animal models, it has been implicated in a variety of functions, including circadian timekeeping through its chrono-modulatory properties [29,30,31,32]. This hormone prevents weight gain in many experimental animal models [29,33], although there is no clear consensus among studies regarding melatonin’s ability to promote weight loss [29,34,35,36]. Currently, its role in the regulation of lipid metabolism in various tissues is being analyzed, with particular attention to the hepatic implications [37,38]. This hormone has generated considerable interest with more than 600 PubMed articles on melatonin and obesity in the last twenty years [33,36,37,38,39]. However, there is no clear consensus on its physiological roles, target proteins or therapeutic applications [40]. This is partly due to the study design (human vs. animal experimental model), timing model or melatonin dose and route of administration [41]. Therefore, further research is needed to elucidate its physiological functions, especially in cellular metabolism.

Therefore, the present study was undertaken to investigate the effects of diet and melatonin on the relative gene expression of lipolytic proteins within the mesenteric adipose tissue and plasma FFA and glycerol levels of male Wistar rats fed a high-fat diet or a control diet. This would facilitate a more comprehensive analysis of the effects of melatonin on cellular adipose tissue metabolism in an experimental model of diet-induced obesity.

## 2. Results

### 2.1. Rhythmicity of Lipolysis Protein Gene Expression and Plasma Glycerol and FFA Levels

Table 1 presents the results of a one-way ANOVA analysis to assess temporal variations in the relative gene expression of each analyzed gene (*Atgl*, *Hsl*, *Cgi58*, *Perilipin*, *Dgat1*, and *Dgat2*) in mesenteric adipose tissue and the plasma concentration of FFA and glycerol in the experimental groups. Additionally, the rhythmicity analysis of these data using CircWave v1.4 software (harmonic regression method with a 24 h period and alpha level set at 0.05) is included.

**Figure 2:**
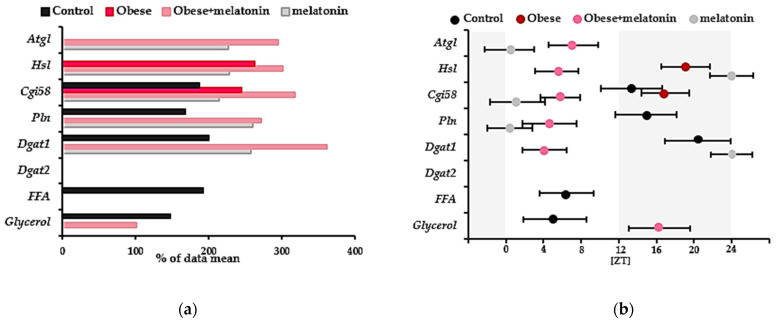
This figure shows the amplitude (**a**) and center of gravity (CoG) (**b**) of *Atgl*, *Hsl*, *Cgi58*, *Perilipin (Pln)*, *Dgat1*, and *Dgat2* gene expression in mesenteric adipose tissue with significant rhythmicity. Additionally, plasma levels of FFA and glycerol are shown. The data are from male Wistar rats fed either a high-fat or a maintenance diet and given one of the following drinking solutions for 11 weeks: tap water or water containing 2.3 mg/kg/day of melatonin. The gray shaded area indicates the dark phase (12:12 h light/dark cycle; lights on at 8:00 AM (ZT0).

The control group, maintained under a 12 h light/12 h dark photoperiod, exhibited significant differences in the relative gene expression of *Cgi58*, *Perilipin*, and *Dgat1* in mesenteric adipose tissue (ANOVA: *p* = 0.006, *p* = 0.044 and *p* = 0.019, respectively). Plasma glycerol and FFA levels also showed significant differences (ANOVA: *p* = 0.0438 and *p* = 0.001, respectively). CircWave analysis further revealed rhythmic patterns in these genes (CircWave: *p* = 0.006, *p* = 0.021 and *p* = 0.01, respectively) and glycerol and FFA plasma concentration (CircWave: *p* = 0.020 and *p* = 0.006, respectively). The center of gravity (CoG) for the relative gene expression of *Cgi58* and *Perilipin* was observed at the beginning of the dark period, while *Dgat1* peaked at the end of this period. Conversely, FFA and glycerol displayed significant daily oscillations with a CoG in the middle of the light period (Figure 2). Individual CircWave curves for each gene and lipolysis product are available in Figure S1 (Supplementary Materials).

However, in the obese group, only the relative gene expression of *Hsl* and *Cgi58* in mesenteric adipose tissue showed significant differences among time points (*p* = 0.049 and *p* = 0.031, respectively). CircWave analysis revealed rhythmic patterns of these genes (*p* = 0.006 and *p* = 0.005, respectively), as described in Table 1. The CoG of *Hsl* gene expression was observed near the end of the darkness photoperiod. Conversely, *Cgi58* gene expression displayed significant daily oscillation with a CoG observed around the middle of this dark period. Therefore, high-fat diet intake likely delayed the observed CoG in the relative gene expression of *Cgi58* in mesenteric adipose tissue compared to the control group and further increased its amplitude, as described in Figure 2. Individual CircWave curves for each gene and lipolysis product are available in Figure S2 (Supplementary Materials).

In the obese + melatonin group, relative gene expression of *Atgl*, *Hsl*, *Cgi58*, *Perilipin*, and *Dgat1* in mesenteric adipose tissue, as well as plasma glycerol levels, exhibited significant differences among time points (*p* = 0.0003, *p* = 0.001, *p* = 0.0002, *p* = 0.007, *p* = 0.002, and *p* = 0.018, respectively) and displayed rhythmicity after CircWave analysis (*p* = 0.021, *p* = 0.0002, *p* = 0.00005, *p* = 0.031, *p* = 0.029 and *p* = 0.040), as described in Table 1. The CoG of *Atgl*, *Hsl*, *Cgi58*, *Perilipin*, and *Dgat1* relative gene expression was observed in the middle of the light photoperiod. However, plasma glycerol concentration showed significant daily oscillation with a center of gravity observed in the early hours of the dark photoperiod. Therefore, melatonin treatment in obese animals advanced the CoG of the relative gene expression of *Cgi58, Perilipin and Dgat1* and, while it delayed the CoG of plasma glycerol concentrations, as compared with control group. Interestingly, melatonin treatment also advanced the CoG of *Hsl* and *Cgi58* gene expression observed in untreated obese animals. Furthermore, in the obese + melatonin group, the amplitude of genes exhibiting rhythmicity is greater than that described in the control group or in the obese group. However, the amplitude of plasma glycerol concentration is lower compared to the data obtained for the control group, as described in Figure 2. Individual CircWave curves for each gene and lipolysis product are available in Figure S3 (Supplementary Materials).

In the melatonin group, kept in a photoperiod of 12 h of light and 12 h of darkness, the relative gene expression of *Atgl, Hsl*, *Cgi5*, *Perilipin* and *Dgat1* in mesenteric adipose tissue, showed differences among time points (*p* = 0.017, *p* = 0.016, *p* = 0.012, *p* = 0.001 and *p* = 0.01, respectively) and exhibited rhythmicity after data analysis using CircWave (*p* = 0.022, *p* = 0.006, *p* = 0.004, *p* = 0.007 and *p* = 0.0009, respectively), as described in Table 1. Plasma free fatty acid levels did not vary significantly throughout the time points studied, unlike glycerol, whose concentration varied throughout the light and dark photoperiod (*p* = 0.0005). Neither lipolysis products displayed rhythmicity after CircWave analysis. The CoG of the relative gene expression most genes studied in mesenteric adipose tissue from the melatonin group was observed at the beginning of the light photoperiod, as described in Figure 2. Addition, the amplitude of relative gene expression for *Cgi58* and *Perilipin* was significantly higher in the control melatonin group than in the control group. Individual CircWave curves for each gene and lipolysis product are available in Figure S4 (Supplementary Materials).

### 2.2. Melatonin Effects in Lipolysis mRNA Levels in Rat Mesenteric Adipose Tissue Are Diet and Photoperiod Dependent

**Figure 3:**
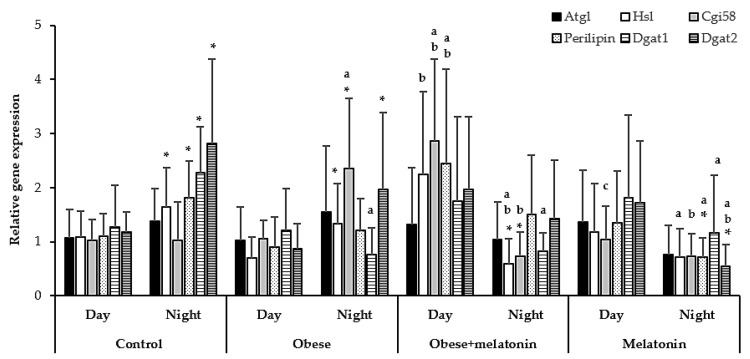
Relative *Atgl*, *Hsl*, *Cgi58*, *Perilipin*, *Dgat1*, and *Dgat2* gene expression in mesenteric adipose tissue of rats fed a maintenance or high-fat diet with tap water or 2.3 mg/kg/day of melatonin in drinking water for 11 weeks. Analyses were performed during light and dark photoperiods. Each value represents the mean ± SD (*n* = 15). Statistical analyses of mean values of mRNA expression of genes were conducted using SPSS Statistics 27.0. If the data met the assumptions of normality, an appropriate statistical test was selected, which could include univariate analysis of variance, independent samples *t*-tests, or one-way ANOVA, followed by post-hoc Bonferroni’s multiple comparison tests to assess group differences. If the data did not meet the normality assumptions, the Kruskal–Wallis test or Mann–Whitney test was employed. * Indicate significant differences between photoperiods within each group *(p < 0.05).* Letters indicate significant differences between diets within the same photoperiod. a = *p* < 0.05 compared to the control group, b = *p* < 0.05 compared to the obese group, c = *p* < 0.05 compared to obese + melatonin group.

Figure 3 shows the effects of photoperiod and diet on mRNA levels of *Atgl, Hsl*, *Cgi58*, *Perilipin*, *Dgat1*, and *Dgat2* in mesenteric adipose tissue in experimental groups.

In the control group, the relative gene expression of *Atgl* and *Cgi58* did not differ according to the photoperiod. However, *Hsl*, *Perilipin*, *Dgat1*, and *Dgat2* gene expression increased significantly during the dark photoperiod compared to the light photoperiod (rest phase) (*t*-tests: *p* = 0.0048, *p* = 0.007, *p* = 0.008 and *p* = 0.023, respectively).

In the obese group, the relative gene expression of *Atgl*, *Perilipin* and *Dgat1* did not differ according to the photoperiod. However, *Hsl, Cgi58* and *Dgat2* gene expression increased significantly during the dark photoperiod compared to the light photoperiod (*t*-tests: *p* = 0.018, *p* = 0.003 and *p* = 0.032, respectively). On the other hand, during the light phase, the obese group showed no significant differences in the relative gene expression of the analyzed proteins compared to the control group. However, during the dark photoperiod, high-fat diet intake increased *Cgi58* expression gene and decreased the mRNA levels of *Dgat1* compared to the data obtained in the control group (One-way ANOVA with Bonferroni post-hoc test: *p* < 0.001; Kruskal–Wallis test: *p* = 0.005, respectively).

On the other hand, in the obese group with melatonin supplementation, *Atgl*, *Perilipin*, *Dgat1*, and *Dgat2* expression did not differ between light and dark photoperiods. However, *Hsl* and *Cgi58* mRNA levels significantly decreased during the dark photoperiod compared to the light photoperiod (Mann–Whitney test: *p* = 0.001; *t*-test: *p* < 0.001, respectively). This group exhibited increased relative gene expression of *Cgi58* and *Perilipin* compared to the control group during the light phase (one-way ANOVA, Bonferroni test: *p* < 0.001, *p* = 0.02, respectively). Melatonin treatment in this group also significantly increased mRNA levels of these genes and *Hsl* compared to the untreated obese group (One-way ANOVA with Bonferroni post-hoc test *p* < 0.001, *p* = 0.006; Kruskal–Wallis test: *p* = 0.021, respectively). However, the effects of melatonin were reversed during the dark photoperiod. The melatonin-treated obese group showed decreased *Hsl* and *Dgat1* gene expression compared to the control group (Kruskal–Wallis test: *p* = 0.008, *p* = 0.023, respectively) and lower *Hsl* and *Cgi58* expression compared to the untreated obese group (Kruskal–Wallis test: *p* = 0.043; one-way ANOVA with Bonferroni post-hoc test: *p* < 0.001, respectively).

In the melatonin group, *Atgl*, *Hsl, Cgi58*, and *Dgat1* expression did not vary according to the photoperiod. However, both *Perilipin* and *Dgat2* gene expression significantly decreased during the dark photoperiod compared to the light photoperiod (*t*-test: *p* = 0.049 and *p* < 0.007, respectively). During the light phase, the melatonin group showed decreased *Cgi58* gene expression compared to the obese group supplemented with melatonin (one-way ANOVA with Bonferroni post-hoc test: *p* < 0.001). In the dark phase, rats fed a maintenance diet and treated with melatonin also displayed decreased relative gene expression of *Hsl*, *Perilipin*, *Dgat1*, and *Dgat2* compared to the control group (Kruskal–Wallis test: *p* = 0.031, *p* = 0.002, *p* = 0.010; and one-way ANOVA with Bonferroni post-hoc test: *p* < 0.001, respectively). Additionally, *Cgi58* and *Dgat2* expression further decreased compared to the obese group (one-way ANOVA with Bonferroni post-hoc test: *p* < 0.001, *p* = 0.037, respectively). 

### 2.3. Melatonin Effects in Plasma FFA and Glycerol Levels in Rats Are Diet and Photoperiod Dependent

**Figure 4:**
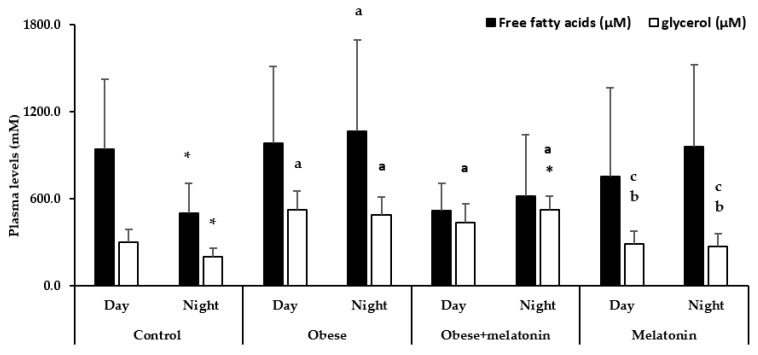
Plasma levels of FFA and glycerol in rats fed with a maintenance or high-fat diet and tap water or 2.3 mg/kg/day of melatonin in drinking water for 11 weeks, analyzed during light and dark photoperiods. Each value represents the mean and standard deviation (SD) (*n* = 15). Statistical analyses of mean values of mRNA expression of genes were conducted using SPSS Statistics 27.0. If the data met the assumptions of normality, an appropriate statistical test was selected, which could include univariate analysis of variance, independent samples *t*-tests, or one-way ANOVA, followed by post-hoc Bonferroni’s multiple comparison tests to assess group differences. If the data did not meet the normality assumptions, the Kruskal–Wallis test or Mann–Whitney test was employed. * Indicates significant differences between the different photoperiods analyzed within each experimental group. On the other hand, the letters indicate significant differences between the type of diet provided within the same photoperiod. a = *p* < 0.05 compared to the control group, b = *p* < 0.05 compared to the obese group, c = *p* < 0.05 compared to the control obese + melatonin group.

Figure 4 shows plasma levels of FFA and glycerol in male Wistar rats as a function of photoperiod and diet. As expected, in rats fed a maintenance diet and tap water, lipolysis products significantly decreased during the dark photoperiod compared to the light photoperiod (*t*-test: *p* = 0.012 and *p* = 0.001, respectively).

However, rats fed a high-fat diet and tap water displayed no significant differences in lipolysis product plasma concentration between photoperiods. Interestingly, during the light photoperiod, the high-fat diet intake in this group increased plasma glycerol levels compared to the control group (one-way ANOVA, Bonferroni test: *p* < 0.001). In contrast, during the dark photoperiod, it increased the concentration of both FFA and glycerol (Kruskal–Wallis test: *p* = 0.040; One-way ANOVA with Bonferroni post-hoc test: *p* < 0.001, respectively).

In rats fed a high-fat diet with melatonin supplementation (2.3 mg/kg/day of melatonin), glycerol levels increased during the dark photoperiod compared to the light period (*t*-test: *p* = 0.021). Interestingly, melatonin treatment decreases towards the control values the dark FFA concentration observed in untreated obese animals, but not statistics significantly. However, melatonin did not prevent the overall elevation of plasma glycerol observed in the obese group in both photoperiods (one-way ANOVA with Bonferroni post-hoc test: *p* = 0.012, *p* < 0.001, respectively).

On the other hand, in the melatonin group, neither FFA nor glycerol levels varied between light and dark photoperiods. Additionally, melatonin treatment in rats fed a maintenance diet did not affect lipolysis product concentrations compared to the control group across both photoperiods. However, the glycerol concentration in the melatonin group was significantly lower than that observed in the obese or obese + melatonin groups during the light (one-way ANOVA with Bonferroni post-hoc test: *p* < 0.001, *p* = 0.006, respectively) or dark photoperiod (one-way ANOVA with Bonferroni post-hoc test: *p* < 0.001, all comparisons).

### 2.4. Morphological and Morphometric Study of the Effects of Melatonin on Rat Mesenteric Adipose Tissue

**Figure 5:**
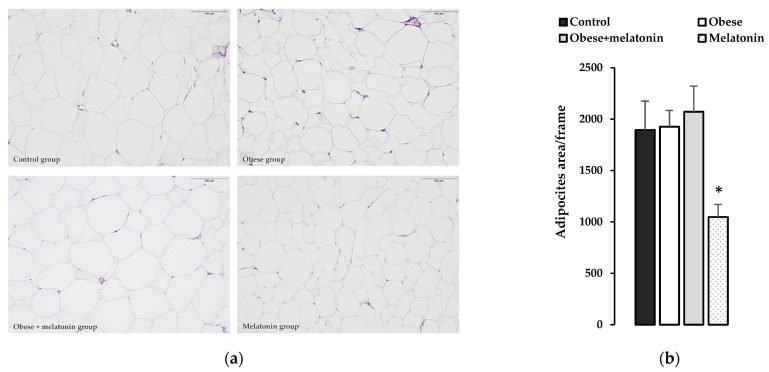
Morphological and morphometrics studies. (**a**) H/E staining of mesenchymal adipose tissue of each of the study group, showing the evident smaller size of mesenteric adipocytes in the melatonin group. (**b**) Data are represented as means ± SD adipocytes area/frame (*n* = 10/12) and significant differences between groups indicated by * *p* < 0.05.

The morphological study of mesenteric fat showed that the adipocytes area in the melanin group observed in each image was significantly lower than in the other groups, implying that the size of these adipocytes is smaller, as shown in Figure 5.

## 3. Discussion

This study showed a daily pattern of relative gene expression in a subset of lipolysis-related proteins in the mesenteric adipose tissue of adult male Wistar rats fed a control diet. Conversely, melatonin administration (2.3 mg/Kg/day) modulated the rhythmicity of lipolysis protein gene expression in rat mesenteric adipose tissue, depending on the type of diet provided.

### 3.1. Rhythmic Gene Expression of Lipolysis Proteins in Rat Mesenteric Adipose Tissue

The suprachiasmatic nucleus (SCN) may regulate cellular metabolism in adipose tissue by synchronizing peripheral clocks, as suggested by murine and human studies [20,24,42,43]. In rat mesenteric adipose tissue, the CLOCK:BMAL1 dimer directly modulates the expression of clock-controlled genes, including *Per* and *Cry*, which peak early in the dark phase [44]. Our findings revealed a circadian pattern only for the gene expression of *Cgi58* and *Perilipin*, with peak expression during the dark phase, coinciding with CLOCK:BMAL1 activity. However, we did not observe a circadian pattern in the relative gene expression of *Hsl* in the mesenteric adipose tissue of adult male rats, consistent with the previous literature [43], although photoperiod-dependent variations in its expression were observed. These results indicate that peripheral clocks may govern circadian activity within adipose tissue through the regulation of the daily expression of specific genes implicated in lipolytic metabolic processes [45], thereby translating temporal information into physiological cues [20,42].

On the other hand, *Dgat1* gene expression displayed a circadian rhythmic pattern with CoG in dark photoperiod (awake phase) that could be associated with the enzymatic function of its protein. DGAT1 synthesizes TAG when there is a high substrate concentration and plays a significant role in protecting the endoplasmic reticulum [43]. *Dgat2* gene expression also increased significantly during the dark photoperiod compared to the light photoperiod, despite not finding a circadian pattern. This enzyme function is related with lipogenesis and TAG synthesis [14]. Therefore, this suggests that the circadian clock could influence the timing coordination of adipose tissue esterification of FFA. Previous studies have shown that the circadian protein PER2 is a direct inhibitor of PPARγ, a key transcription factor involved in adipogenesis and lipid accumulation [44]. However, further research is needed to fully understand the role of the circadian clock in regulating these functions and its potential implications for health.

Lipolysis is activated in the white adipose tissue when insulin plasma levels decrease, resulting in the release of FFA and glycerol into the vasculature for its use by other organs as an energy substrate [45]. In mesenteric adipose tissue, located between the gut and the liver, the lipolytic products are first accessible by the liver [46]. The plasma levels of FFA and glycerol displayed significant daily fluctuations that appear to be contingent not only on rhythmic food intake but also on the proper function of the circadian clocks [24,42]. An important hormone control of lipolysis falls on insulin signaling, that suppresses the metabolic pathway in the adipocyte leading to a reduction in its metabolic products, meanwhile a decrease in insulin level trigger has the opposite effects [45]. Previous data observed that these hormone plasma levels change throughout the day, with the lowest levels observed around 5:00 PM [47]. Therefore, insulin plasma concentration decreases while plasma FFA and glycerol concentration increase (CoG in the middle of the light period). Considering that the light photoperiod is the resting phase of animals, our results are according to the expected mobilization of fat deposit for energy supply. However, as previously described, the relative gene expression of *Cgi58* and *Perilipin* in mesenteric adipose tissue exhibits a diurnal rhythm, with increased expression during the dark photoperiod, in antiphase to the light photoperiod. This anticipatory increase in the expression of lipolysis-related genes promotes metabolic adaptation to recurring feeding-fasting cycles [18].

### 3.2. Effect of HFD on Daily Expression of Lipolysis Genes in Rat Mesenteric Adipose Tissue

Obesity or excessive weight gain is the result of an imbalance between energy intake and expenditure [48]. Diets rich in fat intake could cause obesity, hyperglycemia, insulin resistance and increase lipolysis in animals [44,47,49]. In mice fed with HFD, it is observed that mesenteric adipose tissue metabolic activity increases more than other visceral depots, and this response may protect the liver from lipid toxicity [46].

In addition to these metabolic effects, this type of diet has been found to disrupt circadian patterns in the plasma concentrations of hormones such as insulin and adipocytokines, as previously described in rats [47,50]. Our results also demonstrate that HFD intake causes significantly circadian disruption in the relative gene expression of the analyzed genes as well as in FFA and glycerol plasma levels. The nutrient components and its availability are key regulator of circadian clocks, mainly in the liver [16,51,52]. HFD induces rapid reprogramming of gene transcription rhythms before overt increases in adiposity in mice [53]. However, the influence of a high-fat diet on adipose tissue biological rhythms remains less understood. Xin et al. (2022) found that HFD disrupted the rhythmic expression of the *Clock* gene in adipose tissue of mice, which is restored by switching to a low-fat diet [16]. However, further studies are warranted to elucidate the effects of diet on the circadian clock in rat adipose tissue and its disruptive effects on adipocyte function.

Additionally, the relative gene expression of *Dgat1* in the dark photoperiod decreased in obese rats, which could reduce DGAT1 protein availability for TAG synthesis and increase lipid-induced endoplasmic reticulum stress, as compared with control group [54]. However, high-fat diet intake did not alter the relative gene expression of *Dgat2*, which could contribute to maintaining DGAT2 protein availability for TAG synthesis and lead to lipid droplet expansion during obesity [55,56].

In obese rats, FFA and glycerol concentrations increased during the dark photoperiod (active/wakefulness phase), reaching similar levels to those observed during the light phase. Therefore, lipolysis is dysregulated in obese animals, with an expected increase during the active, feeding period. Notably, in this diet-induced obesity (DIO) model, despite observed increases in plasma insulin levels during the light photoperiod [33,47], there is no corresponding decrease in plasma lipolysis products. Insulin is the most potent antilipolytic hormone and acts rapidly; rat plasma FFA levels are suppressed by ~90% within 5 min of raising insulin to postprandial levels [19].

Therefore, collectively, these findings suggest chronodisruption, hyperinsulinemia, and peripheral insulin resistance in this DIO model, consistent with previous findings [4,57].

### 3.3. Effects of Melatonin on the Daily Expression of Lipolysis Genes in Mesenteric Adipose Tissue of Rats Fed a High-Fat Diet or Control Diet

Melatonin is a reliable circadian time-giver to brain and peripheral structures expressing melatonin receptors, such as adipose tissue [32,58]. In our study, melatonin treatment (2.3 mg/Kg/day) did not prevent the disruption of relative gene expression throughout the day in the analyzed genes in mesenteric adipose tissue, associated with high-fat diet intake. However, *Atgl*, *Hsl*, *Cgi58*, *Perilipin*, and *Dgat1* relative gene expression in both melatonin-treated animal groups displayed a circadian pattern, different from the control group. The acrophase values for the relative gene expression of these proteins differ depending on the diet provided. Melatonin could modulate the gene transcription of these proteins by regulating clock genes transcription in adipose tissue, such as *Clock* and *Bmal1*, as suggested in vitro studies [59], in experimental animals models with other treatments (20 mg melatonin/Kg/day) [36,60] or animals pinealectomy [61].

The effects of melatonin on the gene expression of this study are observed during the dark photoperiod (active/wakefulness phase) in both treated animal groups. In this photoperiod, melatonin treatment (2.3 mg/kg/day of melatonin in tap water) decreased relative gene expression of *Hsl* in mesenteric adipose tissue, as compared with the control group, and regardless of the diet provided. However, other authors describe that melatonin promotes lipolysis via upregulation of *Hsl* gene expression in mouse fibroblasts [62] and other genes such as *Perilipin* in bovine intramuscular adipose tissue [58]. Therefore, further studies are still needed to analyze the effect of melatonin on the relative gene expression of this enzyme. In addition to regulating *Hsl* gene expression, it could participate in controlling the gene transcription of other proteins such as CGI58, which is regulated by feeding and fasting cycles and by diet type, such as high-fat diets [63], but for other factors, its participation in controlling the gene transcription should be analyzed [62].

Our results indicate that melatonin treatment reduces the expression of key genes involved in triglyceride synthesis in mesenteric adipose tissue. Specifically, we observed a decrease in the expression of *Dgat1* in both high-fat diet and control diet groups, as well as a reduction in *Dgat2* expression in the melatonin group. This suggests a decreased capacity for triglyceride synthesis, potentially due to the reduced availability of FFA for storage. These findings align with previous research by De Farias et al. (2019), which showed that TAG synthesis in mouse subcutaneous adipocytes is downregulated in obese mice treated with melatonin, accompanied by a decrease in *Dgat2* expression [64]. Furthermore, our study in rats fed a control diet and supplemented with melatonin revealed a decrease in adipocyte size and *Perilipin* gene expression in mesenteric adipose tissue, the most abundant coating protein on the surface of the lipid droplet in the adipose tissue [65], along with lower weight gain compared to the control group [33]. These results further support the hypothesis that melatonin may reduce triglyceride storage in adipose tissue.

Numerous studies indicate that melatonin administration can be effective in body mass gain and visceral fat in rodents [32,33,36,66], without changes in the intake, which suggests a negative energy balance that may well be mediated by increased energy expenditure [32,34,36,38] in different tissues such as skeletal muscle, given that the melatonin-treated rats exhibited a significant increase in nocturnal activity [67] or brown adipose tissue activity increased [35]. However, more data are needed to fully understand this effect.

On the other hand, in obese rats, melatonin (2.3 mg/kg/day) prevents the elevation of plasma FFA observed during the dark photoperiod although it does not maintain its rhythmic pattern. However, it does not affect glycerol concentration, as it remains elevated. These results indicate that its effect on lipolysis could also occur at the post-transcriptional level, such as previously has been describe in mice white adipose tissue [68].

Therefore, our data analysis shows the following:

- *Cgi58*, *Perilipin* and *Dgat1* relative gene expression in rat mesenteric adipose tissue and plasma FFA and glycerol levels describe daily rhythms patterns.
- HFD disrupts lipolysis protein relative gene expression rhythms patterns and elevates plasma levels of that metabolism pathway’s products.
- Melatonin administration induces circadian rhythms in *Atgl, Hsl*, *Cgi58*, *Perilipin*, and *Dgat1* relative gene expression in rat mesenteric adipose tissue, with an effect that is diet dependent.
- This treatment prevented fatty acid levels increase during nighttime in obese animals, suggesting its involvement in the regulation of cellular metabolism in rat mesenteric adipose tissue.

Our results confirm the existence of daily rhythmic patterns in the relative gene expression of some of the proteins involved in lipolysis in rat mesenteric adipose tissue. The correct functioning of this pathway requires the existence of these rhythms. These rhythms can be altered by a HFD, highlighting the importance of dietary composition.

A HFD not only has the effect of increasing adiposity, but also acts as a disruptor of these biological rhythms. When considering possible treatments for obesity, not only dietary, but also other aspects such as timing should be considered.

Our results demonstrate melatonin’s role as a chronobiotic molecule regulating circadian rhythms of mesenteric lipolysis protein gene expression and possibly other effects on cellular metabolism. Therefore, its role as a regulator of lipid metabolism should be the subject of further study.

#### Limitations of the Study

The results of this study should be interpreted with caution due to inherent limitations in its experimental design. These limitations include the method of weight gain induction; the administration of melatonin in the drinking water (a single administration precluding the assessment of potential dose-dependent effects); the fixed treatment duration (11 weeks); the sample size at each time point, which may have contributed to greater variability (higher standard deviation) in some data; and the exclusive use of male rats. Moreover, the effects of melatonin at this dosage should be investigated in other anatomical depots with differing biological and metabolic characteristics [1]. These limitations hinder the direct extrapolation of our findings to humans and should be addressed in future studies to improve our understanding of melatonin’s role in the circadian regulation of adipose tissue.

## 4. Materials and Methods

### 4.1. Animals and Experimental Design

This study adhered to the modified ARRIVE guidelines 2.0 for preclinical in vivo research [69] and conformed to the regulatory standards outlined by Spanish and European Union regulations (European Communities Council Directive 86/609/EEC).

Adult male Wistar rats (45 days old, 230–260 g) provided by Envigo (Barcelona, Spain), were housed under standard conditions with a controlled light–dark cycle (12:12 h; lights on at 08:00 AM; ZT0) and temperature (22 ± 2 °C) in the Animal Facility of the Complutense University of Madrid located at the School of Medicine (Registration No: ES-28079-0000086). Following the approval of its protocol by the regional authorities (PROEX184/14) and the Ethical Committee of Animal Experimentation.

**Figure 6:**
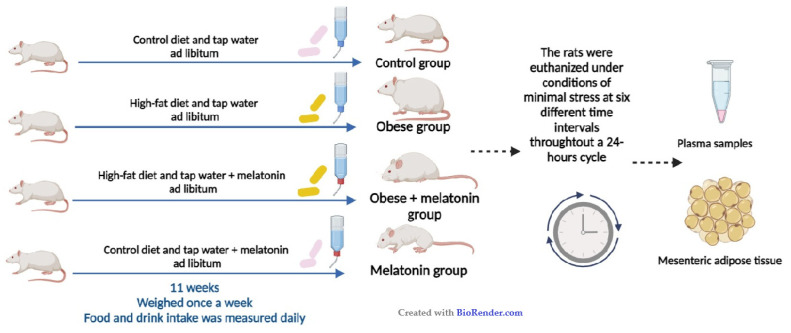
Study design for a diet-induced obesity model in adult male Wistar rats. (Created in BioRender.com, accessed on 20 June 2024).

The study design is reflected in Figure 6. Animals were randomized to four experimental groups resulted from the combinations of a maintenance diet or high-fat diet and tap water or 2.3 mg/kg/day of melatonin (Sigma Aldrich, Madrid, Spain) in drinking water for 11 weeks (*n* = 30 per group). (1) Control group: maintenance diet (control diet) and tap water. (2) Obese group: high-fat diet and tap water. (3) Obese group + melatonin: high-fat diet and melatonin supplementation of 2.3 mg/kg/day in tap water. (4) Melatonin group: control diet and melatonin supplementation of 2.3 mg/kg/day in drinking water. Rats are nocturnal animals; therefore, food intake occurs during the dark photoperiod.

Control diet (4% fat, 60% carbohydrate, 16% protein; 2.9 Kcal/g) and high-fat diet (35% fat, 35% carbohydrate, 20% protein; 5.4 Kcal/g), supplied by Envigo (Barcelona, Spain), have been previously described by our group [33].

The sample size for the described animal model was determined through a power analysis, employing body weight as the primary variable. This strategy was selected because the administration of 2.3 mg/kg/day of melatonin in tap water results in a reduction in body weight gain in rats, which influences adipose tissue adiposity, the subject of the present study. For these calculations, mean and standard deviation values corresponding to control animals and melatonin-treated rats were obtained from Terron et al. (2013) [67]. A parallel group design was assumed, and with a statistical power of 80% and a significance level of 0.05, a sample size of 5 animals per group (per time point) was determined.

Animals had free access to food and water, the intakes of which were measured daily, with water bottles changed every other day. The melatonin dose was chosen based on previous studies [33,36,70,71]. Considering that rats drank about 30 mL per day, with 90–95% of this total daily water intake occurring during the dark period, the daily melatonin dosage used provided approximately 2.3 mg/kg/day of melatonin.

The animals were weighed once a week for 11 weeks. After this period, the rats were euthanized by decapitation under conditions of minimal stress at six different time intervals (*n* = 5 per group) every 4 h throughout a 24 h cycle, starting at 09:00 h: 09:00 (ZT1), 13:00 (ZT5), and 17:00 (ZT9) hours (light photoperiod) and 21:00 (ZT13), 01:00 (ZT17), and 05:00 (ZT21) hours (dark photoperiod). 

Blood samples were collected from the trunk and centrifuged at 1500× *g* for 15 min to isolate plasma. EDTA (6 g/100 mL) was used as an anticoagulant. Plasma samples were then stored at −70 °C until further analysis. Additionally, the mesenteric adipose tissue was dissected rapidly and stored at −80 °C for subsequent analysis.

### 4.2. Real-Time Reverse Transcription Polymerase Chain Reaction (RT-qPCR)

Total RNA extraction was performed using the RNeasy Lipid Tissue Mini Kit and analyzed with the QuantiTec SYBR Green Kit (Qiagen, Hielden, Germany). The iScript™ cDNA Synthesis Kit (Bio-Rad Laboratories SA; Madrid, Spain) was used to synthesize cDNA from 1 μg of total RNA, following to the manufacturer’s protocol. The housekeeping gene β-actin served as a constitutive control for normalization. Reactions were carried out in the presence of 200 nM of specific primers for adipose triglyceride lipase (ATGL), hormone-sensitive lipase (HSL), comparative gene identification-58 (CGI58), Perilipin, diacylglycerol O-acyltransferase 1 (DGAT1) and diacylglycerol O-acyltransferase 2 (DGAT2). Primers were designed using Primer3 software (The Whitehead Institute, https://primer3.ut.ee/, accessed on 18 May 2020), as shown in Table 2.

Real PCR reactions were carried out in an Eppendorf RealPlex Mastercycler (Eppendorf AG, Hamburg, Germany) by 40 cycles of 95 °C denaturation for 15 s, 60 °C annealing for 30 s, and 72 °C extension for 30 s. Detection of the fluorescent product was carried out at the end of the 72 °C extension period.

Serial dilutions of cDNA from the control were used to perform calibration curves to determine amplification efficiencies. For the primers used, there were no differences in transcription efficiencies and the initial cDNA amount in each sample was calculated using the 2^−ΔΔCt^ method [72]. All samples were analyzed in triplicate and in three different measurements. The PCR device automatically calculated fractional cycle at which the amount of amplified target becomes significant (Ct).

To estimate whether treatment or time of day modified the expression of fat β-actin in fat tissue, PCR employing serial dilutions of this housekeeping gene was performed. In this study, Ct did not vary significantly as a function of treatment or of time of day, indicating the validity to employ β-actin as a housekeeping gene.

### 4.3. Biochemical Assays

Plasma-FFA were measured using the Free Fatty Acid Colorimetric/Fluorometric Assay Kit (Abnova, Taipei, Taiwan, KA1666), following the manufacturer’s instructions. In this assay, FFA are enzymatically converted into acyl-CoA and H_2_O_2_, which results in H_2_O_2_ catalyzing a chemical reaction that produces a color change. Briefly, 10 mL of plasma were mixed with 90 mL of working reagent and incubated for 30 min at 37 °C. After incubation, the optical density was measured at 570 nm (550–585 nm). The values of each duplication were averaged and fitted to the standard curve to calculate free fatty acid concentrations.

Glycerol was measured using the EnzyChrom^TM^ glycerol Assay Kit (BioAssay, Hayward, CA, USA, Cat# EGLY-200), following the manufacturer’s instructions. BioAssay Systems’ glycerol assay uses a single working reagent that combines glycerol kinase, glycerol phosphate oxidase, and color reactions in one step. Briefly, 10 mL of plasma were mixed with 100 mL of working reagent and incubated for 20 min at 37 °C. After incubation, the optical density was measured at 570 nm (550–585 nm). The values of each duplication were averaged and fitted to the standard curve to calculate glycerol concentrations.

### 4.4. Sample Preparation for Histological Analysis Using Adiposoft

Tissues were fixed in paraformaldehyde 4% (Sigma Aldrich, Madrid, Spain) and subsequently prepared using standard histological procedures. Two 5 μm sections per block were then stained with hematoxylin-eosin (HE) (Sigma Aldrich, Madrid, Spain) which allowed distinguishing preadipose tissue, cell outlines and nuclei of definitive fat cells using light microscope. Observations and 10–12 high-resolution photographs (20× magnification) per group were performed with a Nikon Eclipse Ci microscope with a camera with NIS Elements F imaging software (Nikon Corp., Tokyo, Japan). All images were stored in uncompressed 24-bit color TIFF format and analyzed by Adiposoft, an automated open-source software for the analysis of adipose tissue cellularity in histological sections (https://imagej.net/plugins/adiposoft, CIMA, University of Navarra, Pamplona, Spain, accessed on 11 July 2024) [73,74].

Using a minimum interval distance of 100 µm will ensure that each section will contain a unique sampling of adipocytes. The inclusion of immature multilocular adipocytes was avoided by excluding cells that were <35 µm in diameter.

To quantify the area of adipocytes per frame, any adipocytes with visible lacerations to the membranes, or the adipocyte boarding the image frame were excluded. Each adipocyte is subsequently labeled with a unique number. The unique adipocyte number is then exported for statistical analysis.

### 4.5. Data Analysis

All data are presented as mean + standard deviation (SD). *p*-values below 0.05 were considered statistically significant.

Variations between time points within individual gene (Atgl, Hsl, Cgi58, Perilipin, Dgat1 and Dgat2) in mesenteric adipose tissue as well as free fatty acid and glycerol in plasma, were assessed using one-way ANOVA. If the data were statistically significant, their rhythmic patterns were analyzed by a harmonic regression method with an assumed 24 h period and alpha level set at 0.05 was employed. This analysis was performed using the CircWave v1.4 software developed by Dr. Roelof A. Hut in Groningen, Netherlands. Statistically significant rhythms were considered when *p* < 0.05. It is important to note that in the presence of rhythmicity, the output from CircWave took the form of one or two sines wave and provided a significant *p*-value. When no rhythmic pattern was detected, the output appeared as a straight line, and no *p*-value was given. In addition to this, CircWave provides the following information: Centre of Gravity (CoG) or the peak phase of the rhythm, representing the general phase of the curve with SD and Circwave F stat, *p*-value and r^2^ [75].

On the other hand, amplitudes of CircWave curves were calculated as percentages of data mean to enable comparison of amplitudes between data sets [difference between the zenith (highest point) and nadir (lowest point) and divided by the data mean (max − min/mean × 100%)].

Statistical analyses of mean values of mRNA expression of in mesenteric adipose tissue, as well as the levels of free fatty acid and glycerol in plasma, during the light and dark photoperiod were conducted using IBM SPSS Statistics 27.0 (IBM Corporation, Armonk, NY, USA). The normality of all evaluated parameters was assessed by Shapiro–Wilk test. If the data met the assumptions of normality, an appropriate statistical test was selected, which could include univariate analysis of variance, independent samples *t*-tests, or one-way ANOVA, followed by post-hoc Bonferroni’s multiple comparison tests to assess group differences. If the data did not meet the normality assumptions, the Kruskal–Wallis test or Mann–Whitney test was employed.

## 5. Conclusions

Our data demonstrate that lipolysis protein relative gene expression in rat mesenteric adipose tissue exhibits daily rhythms patterns. However, a high-fat diet (HFD) disrupts these circadian rhythms and elevates plasma levels of lipolysis products. Melatonin administration (2.3 mg/Kg/day) modulates the circadian rhythms of lipolysis genes in rat mesenteric adipose tissue, depending on diet. Furthermore, this treatment prevents nocturnal increases in plasma fatty acid levels in obese animals, potentially mitigating the adverse effects of ectopic fat deposition. Further research is needed to fully elucidate melatonin’s impact on rat adipose tissue.

## Figures and Tables

**Table 1:** Circadian analysis of relative gene expression in mesenteric adipose tissue and FFA and glycerol in plasma.

Group	Analysis	Data Mean ± SD	ANOVA	CircWave	(CoG ± SD) Zeitgeber Time [ZT]	Amplitude (% of Data Mean)
**Control**	***Atgl*** (mWAT)	1.22 ± 0.57 (relative gene expression)	NS	NS		
	***Hsl*** (mWAT)	1.31 ± 0.677 (relative gene expression)	NS	NS		
	***Cgi58*** (mWAT)	1.02 + 0.56 (relative gene expression)	F = 4.763 *p* = 0.006	F = 4.916 *p* = 0.006 r^2^ = 0.508 *	13.38 ± 3.27	186.27
	***Perilipin*** (mWAT)	1.485 ± 0.66 (relative gene expression)	F = 2.915 *p* = 0.044	**F = 4.704 *p* = 0.021 r^2^ = 0.319**	14.92 ± 3.25	167.68
	***Dgat1*** (mWAT)	1.70 ± 0.94 (relative gene expression)	F = 3.652 *p* = 0.019	**F = 5.736 *p* = 0.01 r^2^ = 0.364**	20.41 ± 3.49	199.41
	***Dgat2*** (mWAT)	1.95 ± 1.35 (relative gene expression)	NS	NS		
	**FFA** (plasma)	772.02 ± 446.70 (μM)	F = 3.086 *p* = 0.0438	**F = 4.910 *p* = 0.020 r^2^ = 0.366**	6.46 ± 2.92	192.57
	**Glycerol** (plasma)	240.4 ± 80.5 (μM)	F = 5.975 *p* = 0.001	**F = 6.253 *p* = 0.006 r^2^ = 0.333**	5.23 ± 3.34	147.46
**Obese**	***Atgl*** (mWAT)	1.30 ± 1.01 (relative gene expression)	NS	NS		
	***Hsl*** (mWAT)	1.02 ± 0.65 (relative gene expression)	F = 2.788 *p* = 0.049	**F = 6.497 *p* = 0.006 r^2^ = 0.382**	19.08 ± 2.60	262.61
	***Cgi58*** (mWAT)	1.76 + 1.16 (relative gene expression)	F = 3.16 *p* = 0.031	**F = 6.660 *p* = 0.005 r^2^ = 0.388**	16.93 ± 2.57	245.13
	***Perilipin*** (mWAT)	1.07 ± 0.58 (relative gene expression)	NS	NS		
	***Dgat1*** (mWAT)	9.72 ± 7.49 (relative gene expression)	NS	NS		
	***Dgat2*** (mWAT)	1.44 ± 1.18 (relative gene expression)	NS	NS		
	**FFA** (plasma)	1033.83 ± 586.04 (μM)	NS	NS		
	**Glycerol** (plasma)	515.6 ± 124.3 (μM)	NS	NS		
**Obese + melatonin**	***Atgl*** (mWAT)	1.13 ± 0.88 (relative gene expression)	F = 7.32 *p* = 0.0003	**F = 4.480 *p* = 0.021 r^2^ = 0.263**	7.19 ± 2.78	294.07
	***Hsl*** (mWAT)	1.44 ± 1.36 (relative gene expression)	F = 5.844 *p* = 0.001	**F = 11.27 *p* = 0.0002 r^2^ = 0.464**	5.42 ± 2.18	300.38
	***Cgi58*** (mWAT)	1.76 + 1.52 (relative gene expression)	F = 7.557 *p* = 0.0002 r^2^ = 0.621	**F = 20.006 *p* = 0.00005 r^2^ = 0.606**	5.78 ± 2.12	317.98
	***Perilipin*** (mWAT)	1.95 ± 1.50 (relative gene expression)	F = 4.182 *p* = 0.007	**F = 3.982 *p* = 0.031 r^2^ = 0.234**	4.60 ± 2.88	271.44
	**Dgat1** (mWAT)	1.31 + 1.24 (relative gene expression)	F = 5.97 *p* = 0.002	**F = 4.178 *p* = 0.029 r^2^ = 0.284**	4.13 + 2.34	360.61
	***Dgat2*** (mWAT)	10.24 ± 7.52 (relative gene expression)	NS	NS		
	**FFA** (plasma)	579.21 ± 341.31 (μM)	NS	NS		
	**Glycerol** (plasma)	481.4 ± 110.2 (μM)	F = 3.492 *p* = 0.018	**F = 3.659 *p* = 0.040 r^2^ = 0.233**	16.325 ± 3.23	100.50
**Melatonin**	***Atgl*** (mWAT)	1.03 ± 0.80 (relative gene expression)	F = 3.555 *p* = 0.017	**F = 4.489 *p* = 0.022 r^2^ = 0.272**	0.37 ± 2.64	227
	***Hsl*** (mWAT)	0.93 ± 0.73 (relative gene expression)	F = 3.64 *p* = 0.016	**F = 6.397 *p* = 0.006 r^2^ = 0.357**	23.97 ± 2.28	228.34
	***Cgi58*** (mWAT)	0.87 + 0.55 (relative gene expression)	F = 3.996 *p* = 0.012	F = 5.254 *p* = 0.004 r^2^ = 0.512 *	1.20 + 2.92	213.56
	***Perilipin*** (mWAT)	1.006 ± 0.75 (relative gene expression)	F = 5.756 *p* = 0.001	**F = 6.060 *p* = 0.007 r^2^ = 0.345**	0.41 ± 2.40	259.64
	***Dgat1*** (mWAT)	1.46 ± 1.31 (relative gene expression)	F = 4.05 *p* = 0.01	**F = 9.651 *p* = 0.0009 r^2^ = 0.456**	23.97 ± 2.19	257.95
	***Dgat2*** (mWAT)	1.12 ± 1.03 (relative gene expression)	NS	NS		
	**FFA** (plasma)	861.53 ± 587.06 (μM)	NS	NS		
	**Glycerol** (plasma)	283.2 ± 82.4 (μM)	F = 6.8 *p* = 0.0005	NS		

This table summarizes the analysis of time-course variations in the expression of individual genes (*Atgl*, *Hsl*, *Cgi58*, *Perilipin*, *Dgat1*, and *Dgat2*) and plasma free fatty acid and glycerol levels within mesenteric adipose tissue. One-way ANOVA identified statistically significant changes over time. Subsequently, statistically significant data were assessed for rhythmic patterns using CircWave v1.4 software kindly provided by Dr. Roelof A. Hut in Groningen, Netherlands l.The absence of r^2^, CoG, and amplitude values indicates the absence of significant rhythmicity, not that of gene expression. NS: Non-significant difference. * The output from CircWave took the form of two sines wave and provided a significant *p*-value. Individual expression curves for each gene, FFA and glycerol plasma can be found in Supplementary Material.

**Table 2:** Sequence of the primers used for real-time PCR.

Gene	Primers
*b-actina*	Forward 5′-ccagatcatgttgagaccttcaa-3′
	Backward 5′-ccagaggcgtacaggatagc-3′
*Atgl*	Forward 5′-ggatgaaggagcagacagct-3′
	Backward 5′-ggcacagacggcagagac-3′
*Hsl*	Forward 5′-aacactacaaacgcaacgaga-3′
	Backward 5′-attcagccccacgcaact-3′
*Cgi58*	Forward 5′-cactgcgacccaagtcatac-3′
	Backward 5′-ttgaactcctctggctggt-3′
*Perilipin*	Forward 5′-atctcctgccaccagacaag-3′
	Backward 5′-gtgctgaccctcctcaaaag-3′
*Dgat1*	Forward 5′-catcggcgggttcttgag-3′
	Backward 5′-tagggaccatccactgctg-3′
*Dgat2*	Forward 5′-gcagcgagaacaagaataaagg-3′
	Backward 5′-ttgagccaggtgacagagaa-3′

## Data Availability

Data are available upon request to the authors at the Department of Biochemistry and Cell Biology, Faculty of Medicine, Universidad Complutense of Madrid.

## Supplementary Materials

The following supporting information can be downloaded at: https://www.mdpi.com/article/10.3390/ijms26020577/s1.
