# Supplementary material for: Daily Lipolysis Gene Expression in Male Rat Mesenteric Adipose Tissue: Obesity and Melatonin Effects
Source: Int J Mol Sci. 2025 Jan 11;26(2):577. doi: 10.3390/ijms26020577 (PMC11765279; doi:10.3390/ijms26020577)
Supplement: Supplementary file 1 [file ijms-26-00577-s001.zip › ijms-3402580-supplementary.pdf]

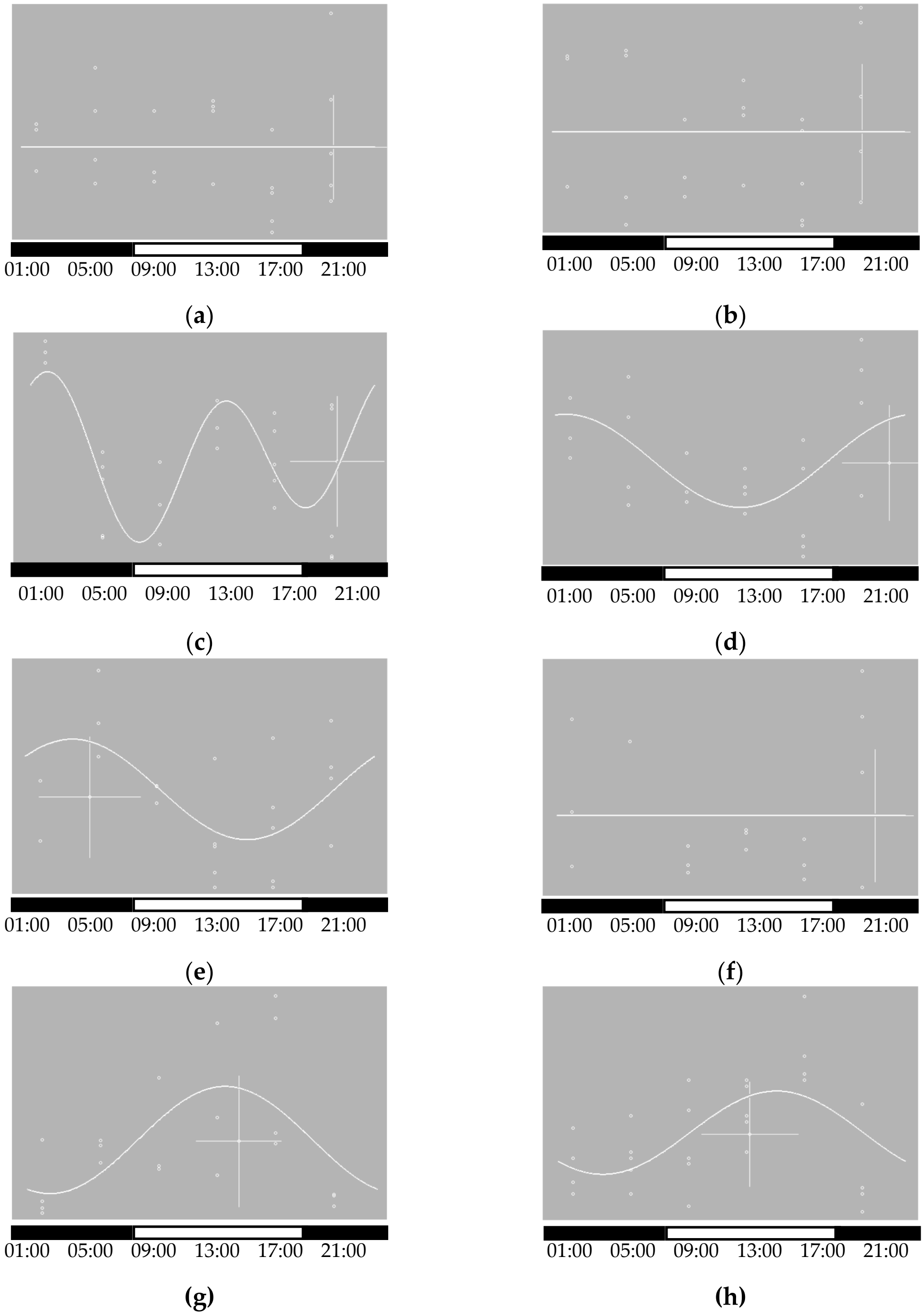

Figure S1. The figure shows the analysis of relative gene expression rhythmicity using CircWave curves for *Atgl* (a), *Hsl* (b), *Cgi58* (c), *Perilipin* (d), *Dgat1* (e), and *Dgat2* (f) in mesenteric adipose tissue from male Wistar rats fed a maintenance diet and tap water. Similar analyses are included for free fatty acid (g) and glycerol (h) plasma concentrations. CircWave v1.4 software, developed by Dr. Roelof A. Hut, utilizes a harmonic regression method with an assumed 24-hour period and an α level set at 0.05. In the presence of rhythmicity, the output takes the form of one or two sine waves and provides a significant p-value. When no rhythmic pattern is detected, the output appears as a straight line.

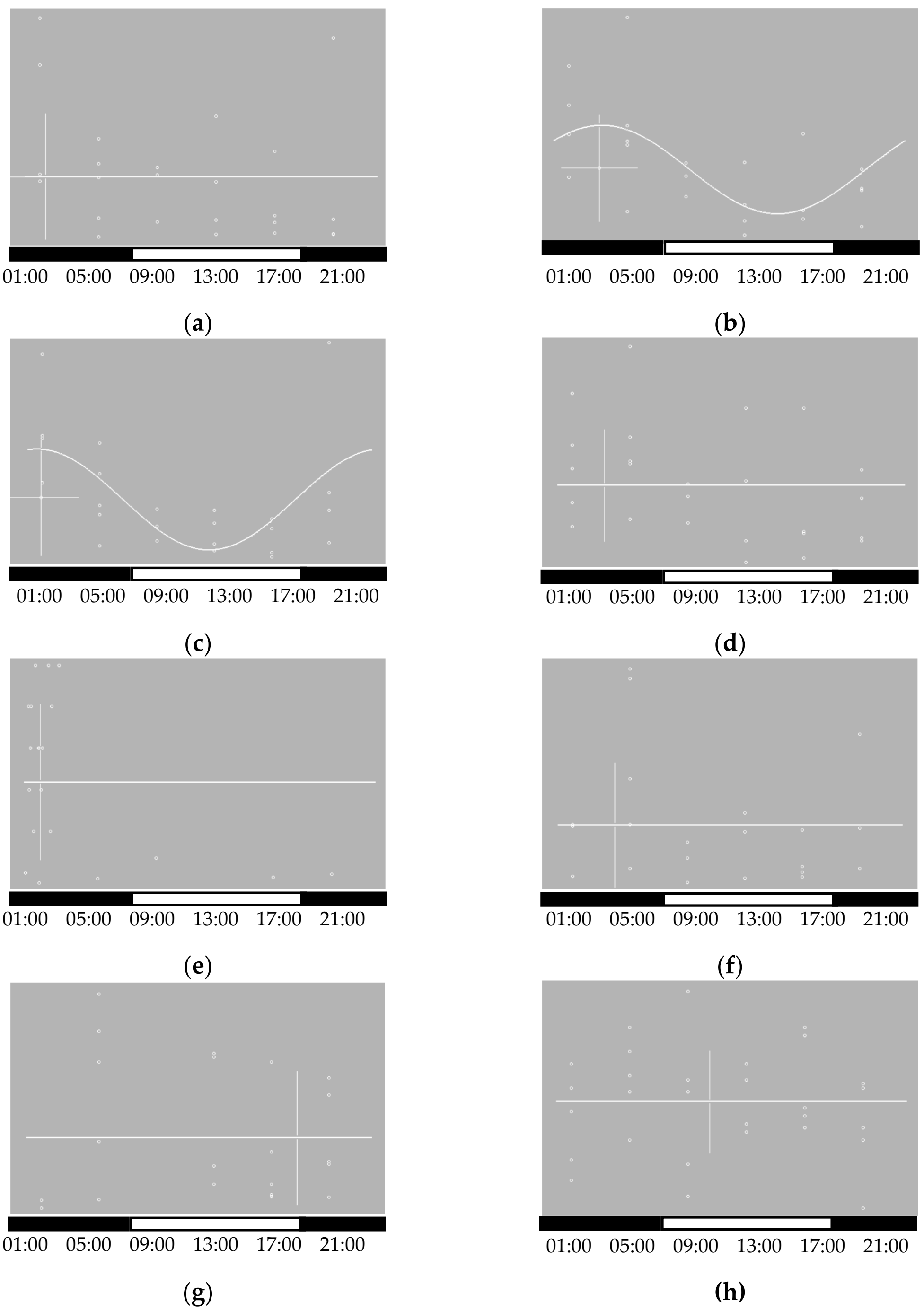

Figure S2. The figure shows the analysis of relative gene expression rhythmicity using CircWave curves for *Atgl* (a), *Hsl* (b), *Cgi58* (c), *Perilipin* (d), *Dgat1* (e), and *Dgat2* (f) in mesenteric adipose tissue from male Wistar rats fed a high-fat diet and tap water. Similar analyses are included for free fatty acid (g) and glycerol (h) plasma concentrations. CircWave v1.4 software, developed by Dr. Roelof A. Hut, utilizes a harmonic regression method with an assumed 24-hour period and an α level set at 0.05. In the presence of rhythmicity, the output takes the form of one or two sine waves and provides a significant p-value. When no rhythmic pattern is detected, the output appears as a straight line.

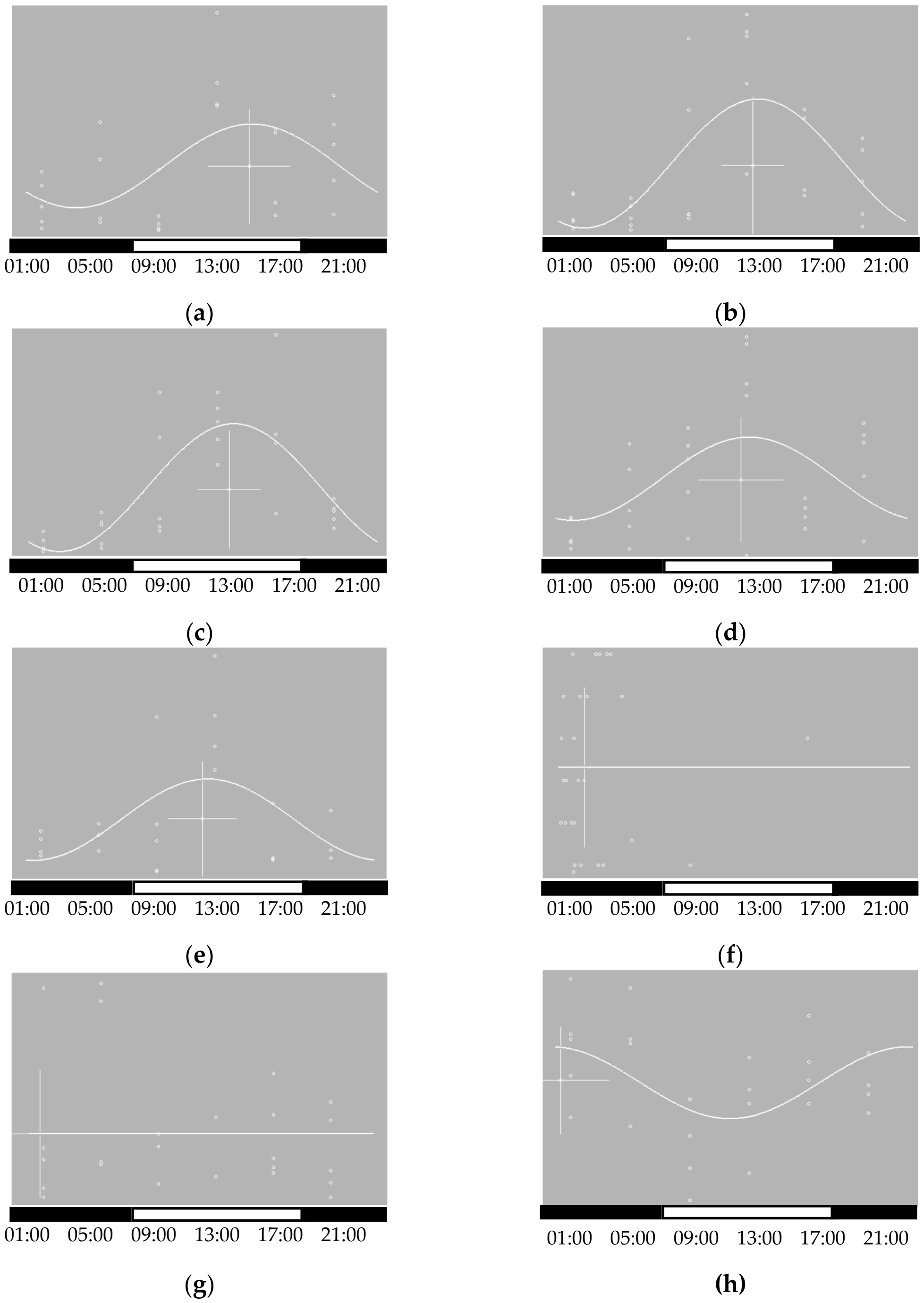

Figure S3. The figure shows the analysis of relative gene expression rhythmicity using CircWave curves for *Atgl* (a), *Hsl* (b), *Cgi58* (c), *Perilipin* (d), *Dgat1* (e), and *Dgat2* (f) in mesenteric adipose tissue from male Wistar rats fed a high-fat diet and 25 μg/mL melatonin in tap water. Similar analyses are included for free fatty acid (g) and glycerol (h) plasma concentrations. CircWave v1.4 software, developed by Dr. Roelof A. Hut, utilizes a harmonic regression method with an assumed 24-hour period and an α level set at 0.05. In the presence of rhythmicity, the output takes the form of one or two sine waves and provides a significant p-value. When no rhythmic pattern is detected, the output appears as a straight line.

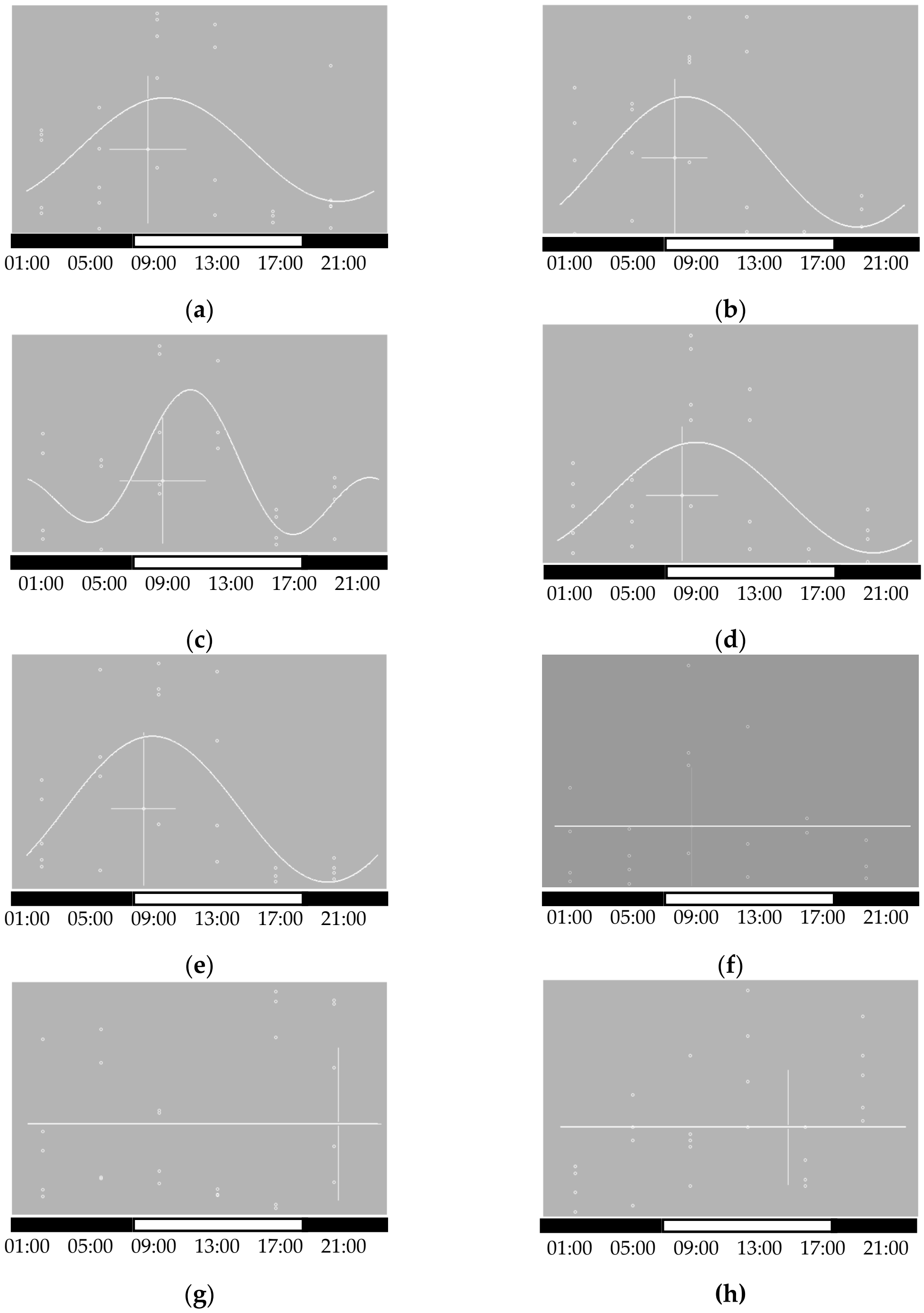

Figure S4. The figure shows the analysis of relative gene expression rhythmicity using CircWave curves for *Atgl* (a), *Hsl* (b), *Cgi58* (c), *Perilipin* (d), *Dgat1* (e), and *Dgat2* (f) in mesenteric adipose tissue from male Wistar rats fed a maintenance diet and 25 µg/mL melatonin in tap water. Similar analyses are included for free fatty acid (g) and glycerol (h) plasma concentrations. CircWave v1.4 software, developed by Dr. Roelof A. Hut, utilizes a harmonic regression method with an assumed 24-hour period and an α level set at 0.05. In the presence of rhythmicity, the output takes the form of one or two sine waves and provides a significant p-value. When no rhythmic pattern is detected, the output appears as a straight line.
